# Immunomodulatory properties of venom-derived crotamine

**DOI:** 10.3389/fphar.2026.1860014

**Published:** 2026-07-23

**Authors:** Isadora Oliveira, Isabela Ferreira, Lorena Sousa, João Alberghini-dos-Santos, Felipe Cerni, Manuela Pucca

**Affiliations:** 1 Medical School, Federal University of Roraima, Boa Vista, Roraima, Brazil; 2 Department of BioMolecular Sciences, School of Pharmaceutical Sciences of Ribeirão Preto, University of São Paulo, Ribeirão Preto, São Paulo, Brazil; 3 Institute of Biomedical Sciences, Federal University of Alfenas, Alfenas, Minas Gerais, Brazil; 4 Department of Clinical Analysis, School of Pharmaceutical Sciences, São Paulo State University, Araraquara, São Paulo, Brazil

**Keywords:** B-defensins, cell-penetrating peptides, crotamine, immunomodulation, NF-κB

## Abstract

Crotamine is a low-molecular-weight cationic polypeptide isolated from *Crotalus durissus terrificus* snake venom, classified as a non-enzymatic toxin with distinctive structural and functional properties. This toxin also exhibits characteristics of a cell-penetrating peptide, owing to its ability to translocate across biological membranes in a largely non-specific manner and to deliver diverse molecular cargos into cells. Beyond its well-established internalization capacity, emerging evidence suggests that crotamine exerts immunomodulatory effects. In our mini review, we discuss current advances in understanding crotamine as a venom-derived immunomodulatory peptide, highlighting its structural similarities with host defense peptides, its interactions with immune cells, and its potential applications in immunotherapy and drug delivery.

## 1 Introduction

Animal venoms constitute a rich source of bioactive molecules, including peptides capable of interacting with different biological targets and modulating physiological processes. Among these compounds, small basic polypeptides present in snake venoms, such as myotoxins, stand out, with crotamine being a representative example (Ownby, 1998). Crotamine is a low-molecular-weight cationic polypeptide characterized by a high proportion of basic residues and a structure stabilized by three disulfide bonds, which contributes to its functional properties and interaction with cellular membranes (Fadel et al., 2005). While crotoxin constitutes the major toxic component of *Crotalus durissus terrificus* venom, crotamine represents a structurally and functionally distinct non-enzymatic peptide (Ownby, 1998). Unlike enzymatic toxins, crotamine is associated with specific biological effects, including myotoxic activity and ability to interact with cells (Ownby, 1998). This myotoxin can be classified as a cell-penetrating peptide (CPP), a group characterized by the ability to cross biological membranes in a largely non-specific manner and to transport different types of molecules, such as proteins, nucleic acids, and drugs, into cells (Kerkis et al., 2004; Gori et al., 2023; Rádis-Baptista and Kerkis, 2011). This property is supported by experimental evidence demonstrating its ability to bind to DNA and promote its internalization into different cell types, highlighting its potential as an intracellular delivery vector (Martiniuk et al., 2021).

In addition to their structural properties and cell-penetrating capacity, several cationic peptides have been widely recognized for their immunomodulatory functions, acting as important regulators of the immune responses. These peptides, often referred to as host defense peptides, play a central role in modulating innate and adaptive immune responses by orchestrating immune cell recruitment, cytokine production, and inflammatory processes. (Hemshekhar et al., 2016). In this context, their activity extends beyond direct antimicrobial effects, including the ability to enhance host defense mechanisms while simultaneously controlling excessive inflammatory responses, thereby contributing to the maintenance of immune homeostasis (Hemshekhar et al., 2016). From a mechanistic perspective, these peptides interact with immune system receptors, such as pattern recognition receptors (PRRs), including toll-like receptors (TLRs), modulating intracellular signaling pathways that culminate in the activation of transcription factors, such as nuclear factor kappa B (NF-κB), and the regulation of cytokine and inflammatory mediator expression (Hemshekhar et al., 2016). In this scenario, molecules with similar structural characteristics, such as crotamine, have attracted increasing interest due to their potential to interact with immune cells and their possible role in modulating inflammatory and immune responses.

Indeed, crotamine has emerged as a molecule of interest due to its diverse biological activities. Structurally, it belongs to the α-myotoxin family and exhibits typical features of cationic peptides, including positively charged regions that favor its interaction with cellular membranes and its ability to penetrate cells (Kerkis et al., 2004; Kerkis et al., 2010; Silvestrini et al., 2019). In addition to its classical effects, such as myotoxicity and neurotoxicity, experimental studies indicate that crotamine is capable of interacting with components of the immune system, modulating the activity of different cell populations, including macrophages, neutrophils, lymphocytes, and mast cells (Silvestrini et al., 2019; Lee et al., 2016; Rodrigues et al., 2013). *In vivo* evidence demonstrates that its administration induces both local and systemic inflammatory responses, characterized by cellular recruitment, increased nitric oxide production, elevated C-reactive protein levels, and concomitant modulation of pro-inflammatory cytokines, such as TNF-α, and anti-inflammatory cytokines, such as IL-10, suggesting a possible regulatory role in the immune response (Silvestrini et al., 2019). However, the molecular mechanisms underlying crotamine-induced immunomodulation remain not fully elucidated, particularly regarding the signaling pathways involved, the specificity of interaction with different cell types, and the balance between pro- and anti-inflammatory effects. Additionally, there is a lack of studies integrating its structural properties, cell-penetrating capacity, and immunological effects, representing a relevant gap in the literature.

Other studies have explored its application as a non-viral delivery system for gene transfer, demonstrating its ability to carry genetic material and promote its internalization into target cells (Martiniuk et al., 2021). Furthermore, recent investigations point to its potential in different approaches, including applications in antitumor therapy and cell targeting, reinforcing its versatility as a molecular tool (Jacob et al., 2026). Therefore, this mini-review aims to highlight crotamine as a versatile multifunctional molecule, summarizing recent advances in its interactions with the innate immune system, its immunomodulatory mechanisms, and its promising therapeutic applications.

## 2 Structure and homology with β-defensins

**FIGURE 1:**
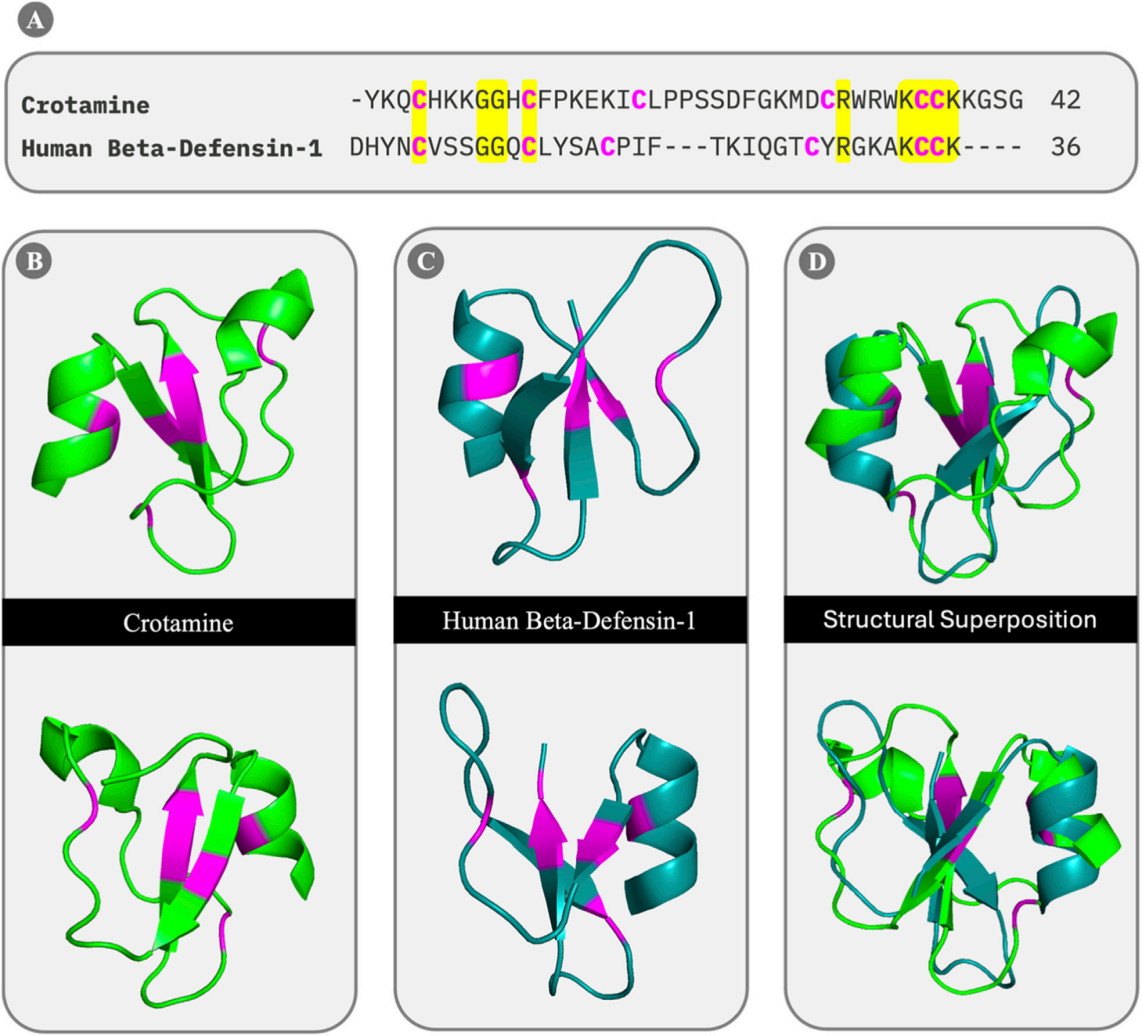
Structural comparison of crotamine (PDB ID: 4GV5 (Coronado et al., 2013a; Coronado et al., 2013b)) and human β-defensin-1 (PDB ID: 1IJV (Hoover et al., 2001; Hoove et al., 2001)). **(A)** Amino acid sequence alignment of crotamine and human β-defensin-1, highlighting conserved residues in yellow. Performed with Multialin (Corpet, 1988). 3D structures of crotamine **(B)** and human β-defensin-1 **(C)** were represented. Three β-strands are similar to both structures, and the overall fold similarity and conserved structural motifs between the two peptides are evident. **(D)** Overlapping structures. Cysteine residues are indicated in magenta on both panels. Molecular models were obtained from RCSB Protein Data Bank (Berman et al., 2000), and optimized with Corel Draw software.

Crotamine is a small, positively charged molecule with a molecular weight of about 4.8 kDa. It consists of 42 amino acids and is rich in arginine and lysine, which gives it an isoelectric point above 9.5. Structurally, it features an α-helix from residues 1 to 9 and an antiparallel β-sheet formed by two strands at residues 9 to 13 and 34 to 38, representing its only defined secondary structures (Coronado et al., 2013a). With six cysteine residues that form three internal disulfide bonds (Cys4-Cys36, Cys11-Cys30, and Cys18-Cys37), its folding closely resembles that of β-defensins, which have a triple-stranded β-sheet with a unique defensin fold (Ganz, 2003) (Figure 1), suggesting a shared gene ancestry despite differences in their primary amino acid sequences. Functionally, defensins exhibit a broad range of activities and produce various effects, with some peptides showing anti-Gram-positive activity and participating in antibacterial defense responses (Kerkis et al., 2014). Crotamine’s defensin-like structure provides a compact shape and a large surface area, allowing it to engage in hydrophobic and electrostatic interactions crucial for its biological roles.

Structural comparison between crotamine (PDB: 4GV5) and human defensin (PDB: 1IJV) revealed significant similarity within their three-dimensional core. Global structural alignment yielded a Root Mean Square Deviation (RMSD) of 2.68 Å over 32 aligned residues, indicating the presence of a conserved structural framework shared by both proteins. Furthermore, alignment based exclusively on the sulfur (SG) atoms of the six cysteine residues involved in disulfide bonds resulted in an RMSD of 0.726 Å, demonstrating a high degree of conservation in the geometry of these linkages (Kufareva et al., 2011). This indicates that crotamine and defensin share a highly conserved disulfide bond-stabilized structural core, whereas the major conformational differences are restricted to peripheral regions of the molecules. The remarkable conservation of cysteine spatial organization suggests that this structural motif plays a critical role in maintaining protein stability and may contribute to the preservation of functional properties common to these cysteine-rich cationic proteins (Betz, 1993; Dias and Franco, 2015). Although they share a similar 3D-fold, the structural homology alone does not establish functional equivalence. Immunological activities of both should be also interpreted cautiously and require direct experimental confirmation.

## 3 Mechanisms of cellular activation

**FIGURE 2:**
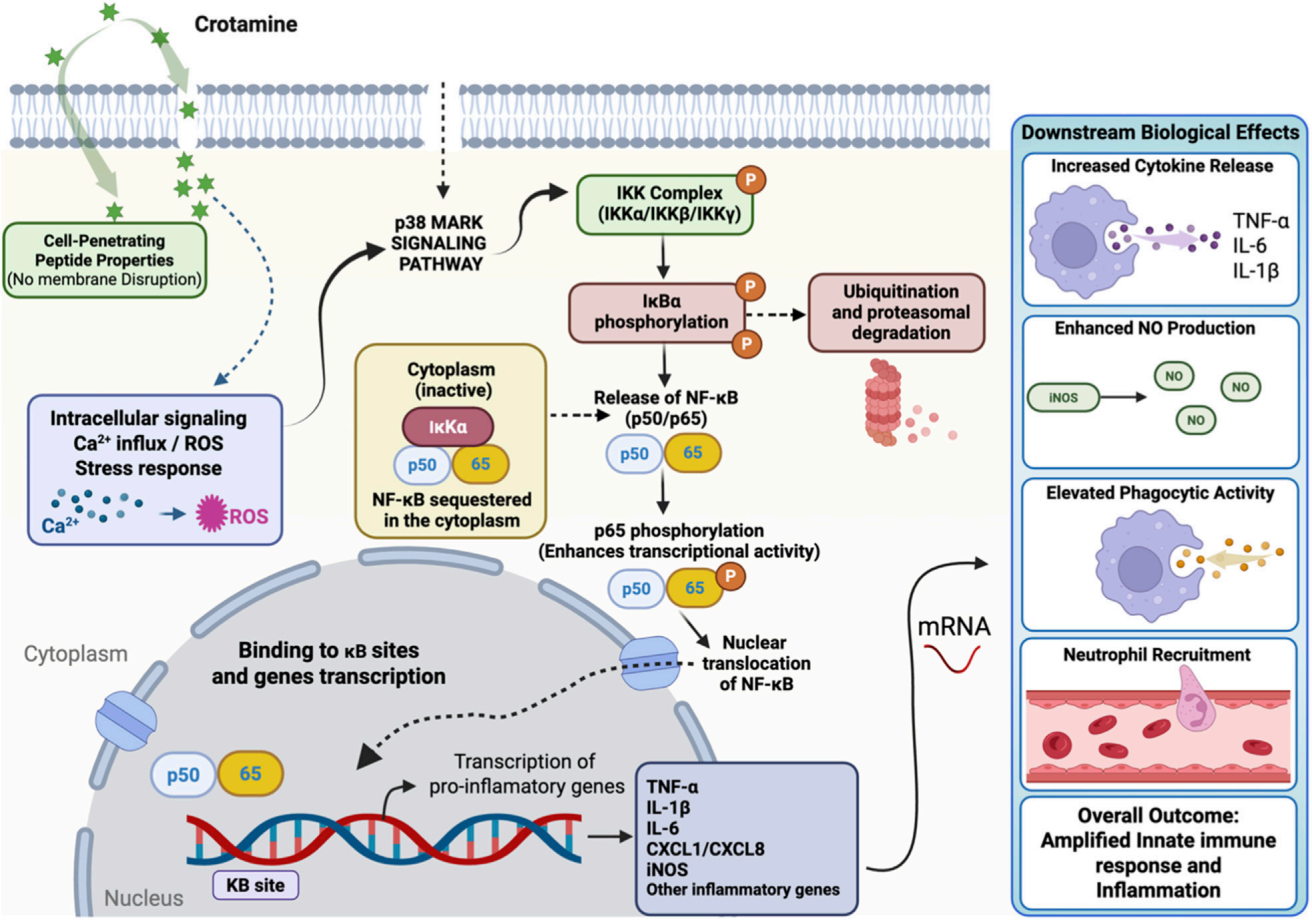
Proposed mechanism of crotamine-induced activation of the p38 MAPK/NF-κB signaling pathway. Crotamine enters immune cells through its cell-penetrating peptide properties without causing significant membrane disruption. Following internalization, crotamine induces intracellular signaling events, including calcium influx, reactive oxygen species (ROS) generation, and cellular stress responses, which promote phosphorylation and activation of p38 MAPK. Activated p38 MAPK stimulates the IKK complex (IKKα/IKKβ/IKKγ), leading to phosphorylation of IκBα and its subsequent ubiquitination and proteasomal degradation. Degradation of IκBα releases the NF-κB p50/p65 heterodimer from its inactive cytoplasmic complex, allowing p65 phosphorylation and NF-κB nuclear translocation. Within the nucleus, NF-κB binds to κB-responsive promoter elements and induces transcription of pro-inflammatory genes, including TNF-α, IL-1β, IL-6, CXCL1/CXCL8, and inducible nitric oxide synthase (iNOS). The resulting increase in inflammatory mediator production contributes to enhanced cytokine secretion, nitric oxide generation, increased macrophage phagocytic activity, and neutrophil recruitment to sites of inflammation, collectively amplifying the innate immune response. The signaling pathway shown represents a proposed mechanistic model integrating the biological effects observed between p38 MAPK and NF-κB signaling pathways (Ma et al., 2024; Cuadrado and Nebreda, 2010). Figure created with BioRender.com.

A distinctive property of crotamine is its ability to penetrate cellular membranes without inducing considerable cytotoxicity, a feature characteristic of CPPs (Kerkis et al., 2004). This property enables intracellular interactions that trigger signaling pathways associated with inflammatory activation, evidencing its function as an immunomodulatory component, in particular innate immune cells, i.e., crotamine modulates transcriptional programs in immune cells, particularly in macrophages (Silvestrini et al., 2019). This section outlines the mechanisms that are currently known to mediate crotamine’s biological activity (Figure 2).

### 3.1 Signaling pathways

The immunomodulatory activity of crotamine appears to involve the activation of intracellular signaling cascades associated with inflammatory responses. Lee et al. (2016) suggests that the exposure of cells to crotamine induces the phosphorylation of p38 mitogen-activated protein kinase (p38 MAPK), a signaling molecule that plays a central role in translating extracellular stress signals into transcriptional responses. This pathway activation is accompanied by stimulation of the NF-κB signaling axis. Specifically, crotamine treatment has been associated with the nuclear translocation of the NF-κB p65 subunit, indicating activation of the canonical NF-κB pathway (Lee et al., 2016).

The coordinated activation of p38 MAPK and NF-κB suggests that crotamine may act as a signaling modulator capable of amplifying inflammatory gene expression. This mechanism contrasts with crotoxin, the major component from *C. durissus* venom that predominantly exert neurotoxic or immunosuppressive effects (Hendon and Tu, 1979; Rangel-Santos et al., 2004). Although the stimulation by crotamine leads to p38 MAPK phosphorylation and NF-κB activation experimentally demonstrated, the upstream molecular targets and the potential involvement of additional signaling pathways remain poorly understood and require further investigation.

### 3.2 Macrophages phenotype

Macrophages exposed to crotamine exhibit functional characteristics consistent with a classically activated, pro-inflammatory (M1-like) phenotype. This activation state is associated with enhanced antimicrobial and inflammatory functions and is typically driven by signaling pathways involving NF-κB activation (Lee et al., 2016; Dorrington and Fraser, 2019). Importantly, it should be noted that macrophage polarization represents a dynamic and context-dependent spectrum rather than a strictly dichotomous M1/M2 classification.

The most notable functional alteration observed in crotamine-treated macrophages is an increase in phagocytic activity. Experimental data demonstrate a higher phagocytic index, indicating an improved capacity of macrophages to internalize microbial particles or cellular debris (Lee et al., 2016). In addition, crotamine stimulation promotes the upregulation of inducible nitric oxide synthase (iNOS) expression. The resulting production of nitric oxide (NO) represents a key effector mechanism of activated macrophages, contributing to microbial killing and participating in the regulation of local inflammatory signaling (Lee et al., 2016).

### 3.3 Cytokines, chemokines and immune cells recruitment

Beyond these mechanisms, crotamine influences the release of soluble inflammatory mediators. The activation of the NF-κB pathway is associated with increased production of tumor necrosis factor α (TNF-α), a cytokine involved in amplifying inflammatory responses and coordinating immune cell recruitment (Dorrington and Fraser, 2019; Osborn et al., 1989). Given the central role of NF-κB in regulating pro-inflammatory gene expression, it is plausible that additional cytokines may also be induced; however, this remains to be experimentally validated in the context of crotamine stimulation.

Also, crotamine appears to promote leukocyte recruitment, particularly of neutrophils. This effect may be partially related to structural similarities between crotamine and β-defensins, due its chemotactic properties. In experimental models, crotamine exposure results in increased neutrophil infiltration, as indicated by elevated myeloperoxidase (MPO) activity, a biochemical marker commonly used to quantify neutrophil accumulation in inflamed tissues (Silvestrini et al., 2019). These findings together indicate that crotamine not only activates resident immune cells, but may also contribute to the orchestration of early innate immune responses through the recruitment of additional effector leukocytes.

## 4 Therapeutic and biotechnological applications

The structural versatility of crotamine is capable of making this myotoxin a major modulator for immune system and, due to this capacity, it can generate factors which limit its therapeutic use, such as inducing an increase in TNF-α and NO levels (Silvestrini et al., 2019; Lee et al., 2016; Alberghini-dos-Santos et al., 2024). However, these effects, combined with the strong cytotoxic factor of the peptide, as well as its potential for cellular penetration, contribute to this compound being a promising candidate for cancer therapy, although its selectivity for cancer cells still needs to be improved (Alberghini-dos-Santos et al., 2024; Hayashi et al., 2020; Jang et al., 2023), since both of the compounds mentioned (NO at high concentrations and TNF-α) may delay cancer progression depending on the cellular context and the type of cell involved (Weigert and Brüne, 2008). However, these responses should not necessarily be interpreted as beneficial immunomodulation, cause the excessive or prolonged activation may trigger the inflammatory imbalance and tissue injury. Thus, the biological significance of these effects is likely to depend on the target tissue, dose, and pathological context.

In addition, crotamine shows potential as an immunomodulatory agent, as it enhances macrophage phagocytic and cytostatic functions through activation of p38 and NF-κB signaling pathways, thereby modulating innate immune responses. These properties may be advantageous in settings such as infection control or tumor immunity; however, their application in immunological disorders requires careful consideration due to the risk of exacerbating inflammation (Lee et al., 2016). Hayashi et al. (2020) demonstrated this potential by showing that crotamine can act as a cellular carrier, forming nanoparticles with DNA and RNA which can deliver genetic material directly into cells that are in active proliferation (Hayashi et al., 2020). In fact, previous studies had already demonstrated that crotamine is a CPP capable of electrostatically binding to plasmid DNA, forming stable complexes, and delivering this genetic material specifically into actively proliferating cells, both *in vitro* and *in vivo*, distinguishing itself from other natural CPPs. This selectivity is possible due to a mechanism involving binding to heparan sulfate proteoglycans on the cell surface, followed by endocytosis and permeabilization of endosomal membranes, allowing the release of the cargo into the cytosol (Nascimento et al., 2007). Complementarily, Jang et al. (2023) showed that crotamine’s selectivity can be increased by producing an innovative recombinant immunotoxin, HER2(scFv)-CRT, which combines crotamine with an antibody fragment directed to the HER2 receptor, increasing specificity and cytotoxicity against breast cancer cells that express this receptor.

Recent studies using human prostate cancer cell line DU-145 show that crotamine effectively blocks cell migration without causing cell death or impacting the viability or migration of non-tumor HUVEC cells (Alberghini-dos-Santos et al., 2024). This anti-migratory effect mainly results from the modulation of voltage-gated potassium channels 1.3 (Kv1.3), which are often overexpressed in prostate cancer and are crucial for membrane polarization (Abdul et al., 2002). These approaches highlight the potential of this myotoxin to expand the repertoire of therapeutic applications, with greater efficacy and selectivity in cancer treatment, by demonstrating that its limitations can be overcome (Jang et al., 2023).

Crotamine is not limited to being a potential anti-cancer agent, but also shows promising action as an antibacterial agent. The study of Oguiura et al. (2011) revealed that crotamine is an antimicrobial peptide (AMP), effective in eliminating three strains of *Escherichia coli* (O15:H7, ML-35p, ATCC 25922), with minimum inhibitory concentrations (MICs) of 25, 50 and 100 μg/mL, respectively, in its native form. In its reduced form, the MIC for these strains was 25, 25 and 50 μg/mL. However, higher concentrations were needed to see some effect against *Pseudomonas aeruginosa* and *Salmonella typhimurium, Listeria monocytogenes* and *Staphylococcus aureus* (Oguiura et al., 2011). Despite this, the myotoxin may also have its activity expanded by presenting activity against the pathogen *Candida albicans* (Youn et al., 2009). This antimicrobial spectrum is linked to the structure of crotamine, which is related to β-defensins (Oguiura et al., 2011; Youn et al., 2009). According to Yount et al. (2009), although both peptides share the ability to disrupt membrane potential and exert antimicrobial effects, crotamine has functionally diverged. In contrast to classical defensins, it preferentially targets and blocks eukaryotic Kv channels, resulting in cytotoxic effects and reflecting an evolutionary diversification of its biological functions (Youn et al., 2009).

Recombinant crotamine shows notable pain-relieving and anti-inflammatory effects that appear to act independently of opioid receptors. These effects from intraperitoneal and intraplantar injections are linked to changes in TNF-α levels, indicating that recombinant crotamine could be developed as an agent for pain and inflammation relief, acting at both central and peripheral locations (Park et al., 2021).

Interestingly, the oral administration of crotamine in mice has been shown to induce significant antinociceptive, anti-inflammatory, and antiedematogenic activities, without present toxicity at the tested doses. At the same study, crotamine decreased leukocyte infiltration into inflamed tissues, suggesting an anti-migratory mechanism that may contribute to the regulation of inflammatory responses (Moreira et al., 2021). These observations extending beyond its previously described pro-inflammatory activities.

Research shows that intradermal injection of crotamine in rats triggers strong local and systemic inflammatory responses, along with systemic oxidative stress, similar to effects caused by histamine. The toxin promotes the migration of neutrophils and macrophages, resulting in increased nitric oxide levels and a gradual rise in C-reactive protein (CRP), an important marker of acute inflammation. Additionally, crotamine has a complex immunomodulatory role, elevating pro-inflammatory TNF-α and also boosting anti-inflammatory IL-10 at lower doses, while concurrently affecting the redox balance and enhancing local angiogenesis through VEGF upregulation. Although its cell-penetrating ability holds promise for drug delivery, these intense inflammatory and immune responses currently restrict its clinical use in its natural form Silvestrini et al. (2019).

## 5 Conclusion and future perspectives

Crotamine emerges as a versatile molecule at the interface between natural toxins and biomedical applications, combining cell-penetrating capacity with immunomodulatory and cytotoxic properties. Within the framework of CPPs, its high cationic charge and affinity for biological membranes support its potential as a vector for intracellular delivery of biomolecules, including therapeutic cargos and vaccine platforms. In addition, crotamine exhibits direct cytotoxic effects against microorganisms and tumor cells, further highlighting its therapeutic application. These features position crotamine as a promising molecule for the development of innovative drug delivery systems, underscoring the broader importance of biomolecules as templates for next-generation therapeutics. However, its clinical translation remains constrained by the need to dissociate beneficial effects on innate and adaptive immune responses from dose-dependent myotoxicity and the risk of systemic inflammatory reactions.

Importantly, the current evidence supporting crotamine-mediated immunomodulation remains limited. Most available studies rely on *in vitro* models and a restricted number of animal studies, while its effects on other immune cell populations and adaptive immunity remain poorly characterized. Moreover, the molecular receptors and upstream signaling events responsible for the immune activation by crotamine have not yet been identified.

Future studies should focus on elucidating its mechanisms of cellular uptake, identifying specific membrane targets, and defining its effects across both innate and adaptive immunity. Also, a particular attention should be given to studies employing human primary immune cells, receptor-blocking approaches, and genetically modified animal models to elucidate the molecular pathways involved in the immunomodulation mediated by crotamine. Further investigations evaluating pharmacokinetics, immunotoxicity, long-term safety, and dose-dependent effects are required to establish its therapeutic window and translational potential. Addressing these gaps will be essential to enable the rational design and preclinical development of safe, targeted, and effective crotamine-based therapeutic applications.
